# “My Colleagues Are the Ones Who Taught Me Everything”: Navigating Workplace Integration Through Organizational Socialization Among Internationally Recruited Nurses: A Qualitative Descriptive Study

**DOI:** 10.3390/healthcare14203471

**Published:** 2026-10-16

**Authors:** Faten Yahya Hassan Alfaifi

**Affiliations:** Department of Nursing, College of Nursing & Health Sciences, Jazan University, Jazan 82911, Saudi Arabia; falfaifi@jazanu.edu.sa

**Keywords:** internationally recruited nurses, organizational socialization, workplace integration, qualitative description, reflexive thematic analysis, nursing workforce, Saudi Arabia

## Abstract

**Highlights:**

**What are the main findings?**

• Workplace integration among internationally recruited nurses was a gradual and uneven process, shaped by the interaction of organizational socialization, workplace relationships, and experiential learning.
• Limited pre-arrival preparation, brief and inconsistently sequenced orientation, and inconsistent mentorship shifted much of the responsibility for learning workplace expectations to nurses and their immediate colleagues.

**What are the implications of the main findings?**

• Healthcare organizations should treat integration as a sustained organizational process that begins before entry and continues through staged onboarding, mentorship, communication support, and supervisory guidance.
• Informal peer learning is valuable but should complement, rather than replace, structured organizational support to reduce variation in integration across clinical units.

**Abstract:**

**Background/Objectives:** International recruitment is used to address nursing workforce shortages, yet recruitment alone does not ensure successful integration into destination healthcare systems. This study aimed to explore how internationally recruited nurses (IRNs) experience and make sense of workplace integration in Saudi Arabia, with attention to the organizational, relational, and experiential processes that shape integration over time. **Methods:** A qualitative descriptive study with reflexive thematic analysis was conducted with 19 IRNs in three tertiary hospitals in southern Saudi Arabia. Participants were purposively recruited, and data were generated through semi-structured interviews. Organizational Socialization Theory (OST) was used as a sensitizing theoretical framework rather than a predetermined coding structure. Data were analyzed using Braun and Clarke’s six-phase reflexive thematic analysis. **Results:** Four interrelated themes captured participants’ integration experiences: (1) Entering the Workplace with Limited Organizational Preparation; (2) Becoming Integrated Through Workplace Relationships; (3) Learning the Unwritten Workplace Through Experience; and (4) Integration as an Uneven Trajectory Over Time. Formal preparation and orientation were often limited, shifting substantial responsibility for workplace learning to peers, supervisors, and experiential exposure. Supportive relationships facilitated learning and belonging, whereas harsh interactions, perceived inequities, and variable organizational support complicated integration. **Conclusions:** Workplace integration was experienced as a gradual, relational, and uneven process rather than a discrete orientation event. Healthcare organizations have a central responsibility for supporting integration, while informal peer learning should complement rather than replace structured organizational support.

## 1. Introduction

Global health systems continue to face nursing shortages that threaten workforce stability and quality of care, prompting greater reliance on international recruitment to sustain service delivery [1,2]. Recruitment alone, however, does not ensure long-term workforce stability. The retention of internationally recruited nurses (IRNs) is influenced by organizational, professional, social, and personal conditions within host healthcare environments, including leadership and supervisory support, working conditions, professional development opportunities, workplace relationships, and organizational recognition [3]. Effective integration is therefore important not only for adaptation to a new healthcare system but also for developing a sense of belonging and a willingness to remain within the host organization [4,5].

Saudi Arabia provides a particularly important context for examining integration processes. Ongoing healthcare transformation, population growth, and expanding service demands have increased the need for a sustainable nursing workforce. Although the national nursing workforce has grown and Saudization initiatives have expanded, Saudi nurses accounted for only 44.22% of the nursing workforce in 2023, indicating continued substantial reliance on IRNs [6]. Recent Saudi evidence also indicates that expatriate nurses experience challenges related to workload, career advancement, leadership opportunities, social and cultural adaptation, and organizational support, all of which may influence retention [7]. This continued reliance makes post-recruitment integration an important workforce-management priority [8]. International recruitment therefore cannot be regarded as complete once nurses cross national borders; destination healthcare organizations must also create conditions that support transition after entry. Such conditions include peer and managerial support, a supportive work environment, professional-development opportunities, effective communication, and organizational practices that facilitate adaptation [4,5]. Workplace integration is consequently better understood as a continuing process through which IRNs learn organizational expectations, establish professional relationships, develop belonging, and adapt to their roles within the host healthcare system.

### Background

Supportive work environments are widely recognized as important for nurse well-being and workforce sustainability [9]. Such environments depend on deliberate organizational structures and practices that support communication, collaboration, leadership, adequate resources, and professional development rather than emerging solely through informal workplace culture. For IRNs, organizational socialization is one mechanism through which these conditions become operational: orientation and support from supervisors and peers can reduce uncertainty and facilitate integration into healthcare organizations [5]. Supportive work environments and organizational relationships are also relevant to job satisfaction and retention [10]. These organizational processes may be particularly important for IRNs, who enter host healthcare systems with professional norms, communication practices, and organizational experiences that may differ from those of the receiving organization. Recent research has identified challenges including language and communication barriers, unfamiliar professional and clinical expectations, cultural adjustment, discrimination, lack of recognition, and difficulties accessing appropriate workplace support [5,11,12]. Conversely, structured orientation and mentorship, together with supportive peer and supervisory relationships, have been identified as facilitators of professional adaptation and workplace integration [5,12].

This study distinguishes three related but analytically distinct concepts: organizational socialization, workplace integration, and professional adaptation. Organizational socialization refers to organizationally shaped processes and tactics through which newcomers acquire the role knowledge, norms, values, and relational understandings needed to participate as organizational members [13].

Workplace integration is conceptualized as a multidimensional and time-dependent process through which IRNs participate effectively in the host healthcare organization, establish meaningful workplace relationships, use and develop their professional capabilities, and foster a sense of inclusion and belonging [14,15]. Professional adaptation refers to the process through which IRNs adjust to differences in professional roles, clinical practices, communication, workplace relationships, and organizational expectations while developing their professional functioning within the host healthcare system [16,17]. In this study, organizational socialization is positioned as an organizational process that may facilitate or constrain workplace integration rather than as a synonym for integration itself. Professional adaptation is treated as one dimension of the broader integration trajectory, reflecting how IRNs adjust their professional functioning while interacting with the social and organizational conditions of the host workplace. Workplace integration therefore encompasses individual adjustment as well as relational and organizational inclusion. Although these processes are interrelated and may unfold concurrently, distinguishing them analytically clarifies how organizational socialization shapes IRNs’ experiences of becoming established members of the host healthcare organization.

Despite growing attention to IRNs’ experiences and integration, the literature has primarily examined transition challenges, cultural and professional adaptation, discrimination, workplace support, and integration outcomes [5,11,18]. Although these studies identify important organizational influences, including orientation, mentorship, leadership, peer support, and workplace relationships, less attention has been given to how formal and informal processes operate collectively as mechanisms of organizational socialization through which IRNs learn organizational expectations, develop workplace relationships, and progressively become established members of host healthcare organizations.

Within Saudi Arabia, research involving IRNs has primarily focused on cultural adaptation and cultural competence, migration and retention challenges, workplace support needs, and, more recently, mentorship and professional belonging [7,19]. Recent Saudi evidence has shown that IRNs experience challenges related to workload, career development, leadership opportunities, social adaptation, and organizational support [7], while emerging research suggests that mentorship may facilitate cultural navigation, understanding of organizational norms, professional networking, and organizational belonging within multicultural Saudi healthcare settings [20]. These factors have generally been examined as discrete challenges or retention-related experiences rather than as interconnected components of an organizational socialization process. Viewed through an organizational socialization lens, these challenges can be examined as features of how IRNs are prepared, supported, and integrated across organizational entry and later workplace adjustment. Consequently, limited evidence explains how formal and informal socialization interact across pre-entry preparation, organizational encounter, mentorship and other socialization agents, informal peer learning, acquisition of workplace norms, and the progressive development of workplace integration over time. By tracing these processes across the integration trajectory of IRNs, this study provides a process-oriented account intended to inform more coherent pre-arrival preparation, staged orientation, mentorship, and supervisory support in Saudi hospitals while contributing destination-country evidence to the broader literature on international nurse mobility.

The purpose of this study was to explore IRNs’ experiences of workplace integration in Saudi Arabia, focusing on how organizational, relational, and experiential processes influenced their integration. This study therefore addressed the following research question: How do IRNs experience workplace integration in Saudi Arabia, and what organizational, relational, and experiential factors shape this process over time?

## 2. Materials and Methods

### 2.1. Study Design

This study employed a qualitative descriptive design with reflexive thematic analysis (RTA) as the analytic approach. Qualitative description was selected because the study sought a practice-near account of IRNs’ experiences of workplace integration in Saudi healthcare settings without attempting to generate formal theory, identify an essential structure of lived experience, or produce an ethnographic account [21]. RTA was selected because it provides a flexible approach for developing patterns of shared meaning across a dataset while explicitly recognizing the researcher’s active role in interpretation and theme development [22]. The study therefore combined a descriptive orientation with an interpretive analytic process: participants’ accounts remained central, while analysis moved beyond topic summary to develop coherent themes relevant to the research question. Organizational Socialization Theory (OST) was used as a sensitizing theoretical framework rather than as a predetermined coding template. The study was reported in accordance with the Standards for Reporting Qualitative Research (SRQR) [23].

### 2.2. Theoretical Framework

Organizational Socialization Theory (OST) provided the theoretical orientation for understanding how IRNs learn to function within a new organizational and professional environment. Organizational socialization concerns the processes through which IRNs learn the roles, norms, behavioral expectations, and organizational understandings needed to function as organizational members [13]. A complementary resource perspective emphasizes newcomers’ access to social, developmental, and organizational resources that support adjustment during socialization [24]. Two complementary perspectives within the organizational socialization literature informed the study. Feldman’s [25] stage model conceptualizes socialization as unfolding through anticipatory socialization, encounter, and change and acquisition, thereby providing a temporal lens for considering newcomer experiences before organizational entry, during initial workplace exposure, and through subsequent adjustment. In contrast, Van Maanen and Schein’s [13] socialization tactics provide a structural perspective on how organizations shape newcomer learning and adjustment through different approaches to orientation, sequencing of learning experiences, role modeling, and access to experienced organizational members. Together, these perspectives enabled consideration of both when socialization experiences occurred and how they were organized and supported. OST served as a sensitizing framework rather than a prescriptive explanatory model. It informed the development of the interview guide by directing attention to areas such as pre-entry preparation, orientation, mentorship and other socialization agents, peer and supervisory support, workplace learning, and acquisition of organizational and cultural norms, without prescribing categories for analysis. Workplace integration was conceptualized more broadly than organizational socialization alone, encompassing participants’ relational, professional, communicative, and contextual experiences within the host workplace. After theme development, Feldman’s stages and selected socialization tactics described by Van Maanen and Schein were used as interpretive concepts to examine the timing, structure, sequencing, and relational support reflected in participants’ accounts. The framework did not determine codes or themes, and accounts that did not align with OST concepts were retained rather than forced into the theoretical model.

### 2.3. Setting and Participants

The study was conducted in three large tertiary hospitals in the southern region of Saudi Arabia. These hospitals function as major referral centers and provide specialized services across a range of clinical areas. They were selected because they employ substantial numbers of IRNs and provided access to nurses working across varied clinical environments. Eligible participants were IRNs who were licensed to practice in Saudi Arabia, had completed at least one year of employment in a Saudi healthcare institution, were engaged in direct clinical care, and were able to communicate effectively in English, the common professional language used in the participating hospitals. The one-year employment criterion was intended to ensure that participants had sufficient exposure to organizational orientation, workplace relationships, communication practices, and clinical-unit expectations to describe their workplace integration experiences. Healthcare professionals from disciplines other than nursing were excluded.

### 2.4. Sampling and Recruitment

Purposive sampling was used to recruit IRNs with direct experience of workplace integration in Saudi healthcare settings. Recruitment across different clinical units sought variation in clinical setting, organizational support, and professional experience rather than statistical representation. Formal coordination with nursing administration departments facilitated initial access to the study sites. Nursing administrators and head nurses facilitated logistical access but were not involved in identifying, nominating, or selecting individual participants. Eligible IRNs received an information sheet explaining the study purpose, eligibility criteria, voluntary nature of participation, confidentiality procedures, and contact information. IRNs who wished to participate contacted the study interviewers directly, and interviews were scheduled at mutually convenient times. Nineteen participants were included. Participation was voluntary, and the final purposive sample comprised the eligible nurses who elected to participate; it was not intended to represent the national nursing workforce.

Previous literature identifies the Philippines, India, and Malaysia as predominant sources of expatriate nurses in Saudi Arabia, with Filipino and Indian nurses representing substantial groups within the expatriate nursing workforce [26]. Recent national statistics provide additional workforce context, indicating that approximately 76% of nurses were female and 24% male, while non-Saudi nurses represented 56% of the workforce compared with 44% Saudi nurses [27]. Sample adequacy was assessed in relation to the information power of the dataset. The focused study aim, specificity of the participant group, use of organizational socialization theory as a sensitizing framework, and the relevance and variation of participants’ accounts supported the adequacy of the 19 interviews for addressing the research question [28]. Consistent with reflexive thematic analysis, adequacy was judged by the richness and complexity of the data rather than by a fixed numerical threshold or a claim of data saturation [29].

### 2.5. Data Collection

Data were collected through individual, face-to-face semi-structured interviews conducted between September and December 2025. Interviews were conducted in English in private locations within the participating hospitals. The interview guide was developed by the author based on the study aim, relevant literature on IRNs’ workplace integration and professional adaptation, and sensitizing concepts from Organizational Socialization Theory (OST). Open-ended questions addressed pre-arrival preparation, orientation, workplace support, communication, professional adjustment, clinical-team relationships, cultural adaptation, and workplace integration. OST oriented attention to potentially relevant organizational processes but did not restrict participants to predetermined theoretical categories; follow-up probes allowed participants to elaborate on experiences and meanings beyond those anticipated in the guide. The guide was pilot tested with two IRNs for clarity, relevance, and sequencing. Minor revisions were made to improve question flow, and the pilot interviews were excluded from the final analysis.

The interviews were conducted by two nursing faculty members, each holding a PhD and having experience in qualitative interviewing. Neither held a supervisory or managerial relationship with participants. The interviewers followed a semi-structured interview guide. The interviews were conducted at times convenient for participants and lasted approximately 15–25 min. Participants were encouraged to elaborate on their experiences, and probing questions were used when necessary to obtain more detailed information. Because questions concerning pre-arrival preparation and early workplace integration required participants to recall earlier experiences, participants were encouraged to provide specific examples and clarify the context and sequence of recalled events. All interviews were audio-recorded and transcribed verbatim by the interviewers. The author subsequently checked each transcript against the corresponding audio recording to verify accuracy and completeness before analysis. Identifying information was removed from the transcripts before analysis, and participants were assigned study codes to protect confidentiality.

### 2.6. Data Analysis

Data were analyzed using Braun and Clarke’s six-phase reflexive thematic analysis (RTA) within the qualitative descriptive design [22]. Microsoft Excel was used as an organizational tool to manage interview excerpts, evolving codes, candidate themes, thematic maps, and reflexive notes; the software did not automate coding or theme development. The analysis was conducted by the author and was inductively driven at the level of coding, with Organizational Socialization Theory (OST) used only at the level of later interpretation rather than as a predetermined coding framework.

Phase 1: Familiarization. The author became familiar with the dataset through repeated reading of the interview transcripts and review of the audio-recorded interviews. Initial engagement with the data focused on gaining an overall sense of participants’ accounts before formal coding began. During subsequent readings, preliminary observations were noted regarding recurring issues, potentially meaningful passages, and emerging patterns related to workplace integration. Reflexive notes were also maintained to document the author’s developing interpretations, assumptions, and reactions to the data. Particular attention was given to accounts of pre-arrival preparation, early organizational entry, workplace relationships, communication, and adjustment over time. These early observations informed later coding and theme development but were treated as provisional rather than as predetermined analytic categories.

Phase 2: Generating Initial Codes. The full dataset was coded systematically to identify data segments relevant to the research question. Concise descriptive or interpretive labels were assigned to meaningful excerpts, with codes kept sufficiently specific to preserve context while allowing comparison across transcripts. Examples included “No pre-departure preparation,” “Minimal organizational orientation,” “Reliance on informal sources,” and “Peer-assisted language learning.” Coding remained primarily semantic and close to participants’ accounts, while some codes captured broader interpretive meanings. Codes were iteratively reviewed, refined, merged, separated, renamed, or discarded as analysis progressed. Consistent with reflexive thematic analysis, no second coder or inter-coder reliability procedure was used.

Phase 3: Generating Initial Themes. Codes were examined for shared patterns of meaning and collated into candidate themes and subthemes relevant to workplace integration. Related or overlapping codes were grouped, while others were repositioned as their analytic relationships became clearer. Through iterative mapping, relationships among codes, subthemes, and emerging themes were repeatedly examined and reorganized as the candidate thematic structure developed. For example, codes concerning limited pre-arrival preparation, brief orientation, and reliance on informal sources contributed to a candidate pattern of constrained organizational entry, whereas codes related to peer support, supervisor approachability, and informal guidance contributed to relational patterns of integration. Candidate themes were actively constructed through interpretation rather than treated as pre-existing categories within the data. An initial thematic map was developed to examine relationships among codes, subthemes, and emerging themes.

Phase 4: Reviewing Themes. Candidate themes were reviewed recursively at two levels: first against the coded extracts to assess coherence within each theme, and then against the full dataset to determine whether the overall thematic structure adequately represented participants’ accounts of workplace integration. Theme boundaries were refined to improve conceptual distinction, particularly where relational conditions of integration overlapped with experiential learning processes. Themes or subthemes that were too broad, weakly supported, or insufficiently distinct were revised, merged, narrowed, or discarded as needed.

Phase 5: Defining and Naming Themes. Each theme and subtheme was given a concise, analytically informative name and a written definition specifying its scope, boundaries, and relationship to adjacent themes.

Phase 6: Producing the Report. The final analytic narrative was developed by integrating participant quotations with the thematic interpretation. After the empirical themes had been established, they were examined in relation to relevant OST concepts. This theoretical interpretation was applied after theme development and did not determine the original codes, candidate themes, or final thematic structure.

### 2.7. Reflexivity

Reflexivity was integral to the analytic process because reflexive thematic analysis recognizes that themes are developed through the researcher’s engagement with the data rather than discovered as objective entities [22]. The author is a Saudi female nursing academic with international academic and professional experience. Her familiarity with the Saudi healthcare and sociocultural context provided contextual knowledge while also creating an insider–outsider position relative to the IRNs. Reflexive notes were maintained throughout coding, theme development, and interpretation to document assumptions, theoretical influences, interpretive shifts, and decisions about theme boundaries.

The interviews were conducted by two female, PhD-qualified nursing academics who were also qualified nurses with prior clinical experience. One interviewer was Filipino and had approximately two years of clinical experience in a surgical intensive care unit in the Philippines; the other was Indian and had approximately one year of clinical nursing experience in India, followed by academic and clinical roles in Saudi Arabia. Their national backgrounds corresponded to the two national groups represented in the participant sample. Shared gender, nursing background, and cultural or migration-related experiences may have facilitated rapport and trust while also creating the possibility of assumed shared understanding. Differences in academic position and doctoral status may likewise have influenced researcher–participant interactions. Open-ended questions, neutral prompts, and clarification were therefore used to encourage participants to describe experiences in their own terms.

Neither interviewer was employed by the participating hospitals or held supervisory authority over participants. Their positionality was considered primarily in relation to data generation, whereas the author’s positionality was considered in relation to analysis and interpretation. Participants’ accounts were treated as situated perceptions of workplace experience rather than objective representations of organizational reality.

### 2.8. Trustworthiness and Methodological Rigor

Methodological quality was approached in a manner congruent with qualitative description and reflexive thematic analysis [21,22]. Credibility was supported through purposive recruitment of participants with direct experience of the phenomenon, open-ended semi-structured interviews with follow-up probing, sustained engagement with the transcripts, reflexive memoing, attention to divergent accounts, and presentation of contextualized participant extracts [22,30].

Dependability and transparency were supported through documentation of the analytic process, including evolving codes, thematic maps, reflexive notes, and records linking participant extracts with developing themes. Confirmability was approached through reflexive examination of the author’s theoretical and professional positioning rather than through claims that researcher influence could be eliminated [22,30]. Developing themes were repeatedly considered against the full dataset, including divergent accounts, and OST-based interpretations were treated as one interpretive resource rather than as evidence that themes were correct [22,30].

Transferability was supported through description of the study setting, participant characteristics, and clinical practice areas, together with sufficiently contextualized participant extracts that enable readers to judge the relevance of the findings to other healthcare contexts [30].

### 2.9. Ethical Considerations

Ethical approval was obtained from the Jazan Health Cluster Ethics Committee, Saudi Arabia (Approval No. 2473; approved 17 October 2024). As the study focused on the workplace, attention was paid to potential power dynamics and perceived coercion. Although recruitment access was facilitated through administrative channels, interested nurses contacted the study interviewers directly. Participation was voluntary, and participants were informed that declining participation or withdrawing from the study would have no professional consequences. Written informed consent was obtained. Interviews were conducted in private settings. Identifying information was removed from the transcripts before analysis, and participants were assigned study codes (P1–P19). Digital data were stored on password-protected devices accessible only to authorized study personnel. Participants were reminded that they could decline to answer individual questions or discontinue the interview at any time.

## 3. Results

### 3.1. Characteristics of Participants

Participant characteristics are presented in Table 1 to provide demographic and professional context for interpreting the findings.

Participant-level contributions to the final themes and the participants represented by illustrative quotations are presented in Supplementary Table S1. Aggregate demographic and professional characteristics are reported in Table 1; detailed participant-level combinations were not presented to minimize the risk of deductive identification.

### 3.2. Thematic Findings

Reflexive thematic analysis generated four interrelated patterns of shared meaning that captured participants’ experiences of workplace integration. These were organized into four themes, with three subthemes identified within Theme 1 (Table 2). The themes should not be interpreted as discrete sequential stages. Rather, they represent interrelated analytical dimensions within a broadly temporal integration trajectory. Theme 1 is most closely associated with pre-entry and early organizational encounter; Themes 2 and 3 describe relational and experiential processes that may operate across the integration trajectory; and Theme 4 captures the uneven development of integration over time.

#### 3.2.1. Entering the Workplace with Limited Organizational Preparation

This theme captured participants’ early experiences of entering and adjusting to the workplace. It reflected how organizational preparation and support shaped their initial understanding of the work environment and their early integration experiences. The subthemes below describe the different ways in which participants encountered and navigated this early stage of organizational entry.

##### Arriving with Limited Workplace-Specific Preparation

Most participants reported receiving minimal structured information about their future workplace before arriving in Saudi Arabia. Participants commonly described receiving little employer-provided workplace-specific preparation. Participants therefore often entered the healthcare system with limited understanding of professional expectations in the host context. One participant described arriving with little formal preparation and instead relying on informal sources to understand the new environment:

“Before coming here, I didn’t get any information. Only my friends who were working here told me about the culture.”

(P1)

She also reflected on how she developed communication skills after arrival:

“No, I did not receive any language training. I learned by talking with patients and their relatives.”

(P1)

When additional information or support was needed, she approached her supervisor:

“If we have any problem, we talk to our supervisor. He is a good person; we can talk with him, and he gives solutions.”

(P1)

Another participant similarly reported that no formal pre-arrival orientation had been provided:

“No, we didn’t have any orientation program before reaching here.”

(P2)

“When I got the offer letter and saw the name of the region, I went to Google it.”

(P13)

Some Filipino participants reported attending Pre-Departure Orientation Seminars (PDOS) organized by their home government. These sessions, however, focused primarily on general cultural expectations rather than workplace-specific preparation.

“We only received information about the place through our Philippine Overseas Employment Administration office (POEA). Regarding the workplace itself, they did not explain it to us. Only during the pre-departure orientation did they explain some cultural expectations, such as wearing an abaya and communicating with Arabic speakers.”

(P14)

These accounts portrayed organizational entry as a period in which participants often had to assemble their understanding of the workplace from informal and external sources. The analytic pattern was not simply an absence of information; it reflected an early transfer of responsibility for preparation from the organization to the newcomer and their personal networks.

Participants also described discrepancies between expectations formed before arrival and the conditions encountered after entry. One participant described unmet expectations regarding accommodation:

“Before coming here, we expected that accommodation would be fully provided by the Ministry of Health. However, when we arrived, we were placed in shared rooms and did not receive what was originally described.”

(P2)

This account illustrates how incomplete or inaccurate pre-arrival information could create expectation–reality discrepancies during anticipatory socialization. Limited workplace-specific preparation before arrival therefore constrained participants’ initial understanding of organizational expectations and increased their reliance on post-entry orientation, peer guidance, and experiential learning.

##### Orientation as a Brief Gateway Rather than a Staged Transition

After arrival, participants commonly described orientation as brief, often lasting approximately one day, and primarily administrative. They rarely addressed clinical expectations or provided staged learning opportunities for gradual role adjustment. One participant described the orientation as follows:

“We had an orientation program conducted by the hospital for one day… within that day the coordinator finished everything.”

(P2)

Following this brief introduction, participants described a limited sense of organizational reception, with little formal welcome or structured support before being assigned to clinical duties.

“They just give an overall description regarding organization policies and protocols. After that we go directly to the workplace… there was no welcome, no support.”

(P2)

Another participant similarly described feeling insufficiently prepared for the clinical environment:

“They are giving only one-day introductions… we don’t know about the hospital situation… so I learned from my colleagues.”

(P3)

Across these accounts, brief orientation functioned less as a gradual transition into practice than as an administrative gateway to the clinical unit. Participants contrasted this with the less common experience of staged preparation and supervised exposure.

“We had three months of training, and only after that they started assigning patients to us.”

(P18)

The contrast between brief orientation and more gradual preparation highlighted substantial variation in how organizational entry was supported. Interpreted through OST after theme development, this variation was consistent with differences in the sequencing and structure of newcomer socialization rather than a uniform organizational pathway.

##### Colleagues Filling the Mentorship Gap

Participants without structured mentorship relied heavily on colleagues and senior nurses to help them understand workplace expectations and clinical routines. Peer interactions therefore functioned as informal socialization mechanisms during early integration.

Participants frequently described learning through observation and everyday interaction with colleagues.

“We learn from our colleagues about the culture.”

(P13)

“With the colleagues only, we are asking what the meaning of this Arabic word is.”

(P2)

Peer guidance was especially important for navigating language and communication challenges.

“Saudi staff taught me the words and how to talk.”

(P1)

These interactions helped participants gradually understand clinical routines, communication styles, and local workplace norms. Participants nevertheless emphasized that this learning occurred informally rather than through institutionalized mentorship. One participant explained:

“Mentorship? I don’t think it’s mentorship… I was supervised by the charge nurse.”

(P11)

Another participant explained that, although IRNs were often expected to draw on their previous experience and quickly adapt to local routines, this did not necessarily constitute mentorship: “Since we are already expats, their expectations [were] really high already. We have experience in the Philippine, so it’s like just… familiarizing [ourselves with] their routines, but not like that mentorship.”

(P11).

Another participant described mentorship as an ongoing form of support for newly recruited IRNs rather than simple oversight:

“Newly hired… mentors or supervisors… be at their back… support them… mentorship means a lot.”

(P14)

Participants also emphasized the importance of having regular opportunities to communicate concerns and receive support. When discussing how this could be facilitated, P14 suggested:

“Monthly meetings… or one on one… hearing them… we want somebody to listen.”

(P14)

When formal mentorship was absent, colleagues and immediate supervisors became the main sources of practical guidance during early integration. Informal relational support enabled learning, but it also made the quality of early integration dependent on who happened to be available and how supportive the local unit environment was. From an OST perspective, these accounts suggest that formal organizational socialization was often supplemented by informal and relational forms of socialization, with experienced colleagues functioning as important socialization agents when structured mentorship was limited.

#### 3.2.2. Becoming Integrated Through Workplace Relationships

A second pattern of shared meaning concerned the relational conditions through which participants developed a sense of acceptance, support, and belonging within the workplace. Supportive colleagues and supervisors provided reassurance, approachability, and interpersonal safety, whereas harsh interactions, blame, and perceived inequities contributed to vulnerability and social distance.

“My colleagues are the ones who taught me everything.”

(P19)

These interactions supported both practical learning and emotional adjustment. Supportive leadership also influenced whether the relational environment felt welcoming and accessible.

“Our head nurse and supervisor were very friendly and professional with me… that made a big difference.”

(P1)

Relational dependence also created vulnerability when workplace interactions were less supportive. Some participants described blame and hierarchical pressure in response to mistakes.

“Some senior staff treat new staff harshly. Even small mistakes receive a lot of blame.”

(P10)

Other participants reported perceived differences between Saudi and IRNs.

“Some differentiation is there… especially in salary and allowances.”

(P5)

Across accounts, workplace relationships functioned as both facilitators and constraints on integration. Supportive interactions strengthened access to guidance, confidence, and belonging, whereas difficult relational experiences could reinforce caution, vulnerability, and distance. Integration therefore involved not only learning how the workplace operated but also navigating one’s position within its social and relational environment.

#### 3.2.3. Learning the Unwritten Workplace Through Experience

A third theme captured integration as learning what was rarely made explicit. Unlike Theme 2, which focused on the relational conditions that facilitated or constrained belonging and support, Theme 3 centered on how participants acquired tacit workplace norms, communication practices, and organizational expectations through observation, repeated exposure, and everyday participation. Experience itself became a major source of organizational learning.

One participant described learning through direct workplace exposure:

“I only became exposed to these things after I arrived and started working.”

(P8)

Language competence and communication practices also developed progressively through workplace interaction.

“We talked with Arabic staff, then I overcame this problem.”

(P7)

Participants described colleagues not primarily as sources of emotional support in these accounts, but as informal sources of tacit workplace knowledge.

“With exposure we can learn some words… my colleagues teach me.”

(P8)

The emphasis here was therefore on experiential knowledge acquisition rather than on the relational quality of collegial support.

Through continued workplace exposure, participants gradually interpreted and adopted implicit workplace expectations. Several described becoming more comfortable navigating the local healthcare environment over time.

“…now I am adapted. I’m used to it. So, I am satisfied.”

(P3)

These accounts portrayed workplace learning as cumulative and experiential. Participants became more able to interpret implicit expectations through repeated participation, but this form of learning also meant that adaptation depended heavily on exposure and access to supportive colleagues rather than on consistently structured organizational guidance. Across these accounts, informal socialization appeared to compensate for gaps in formal preparation, while also making workplace integration more dependent on exposure, local unit practices, and access to supportive colleagues.

#### 3.2.4. Integration as an Uneven Trajectory over Time

The fourth theme captured integration as an uneven trajectory rather than a standardized endpoint. Participants described movement from uncertainty toward greater confidence and familiarity, but the pace and quality of this movement differed according to workplace relationships, organizational conditions, communication demands, and migration-related pressures.

“When I came here, I was very scared… now I know how to stand alone.”

(P13)

Adjustment was not uniform. For some participants, workplace adaptation occurred alongside broader migration-related pressures, including separation from family.

“Sometimes I miss my home and my baby.”

(P7)

Workplace pressures also complicated adjustment for some participants:

“I mean, they’re highly strict, very strict. And their expectations are really too high, and the fact that I am still starting…”

(P11)

These accounts did not support the assumption that time alone led to successful integration. Greater familiarity could coexist with emotional strain, workplace pressure, or continuing experiences of exclusion, indicating that integration remained contingent on the organizational and relational conditions surrounding the nurse.

Participants also described changes in how they perceived the host environment as workplace experiences accumulated. One participant contrasted initial disappointment with later satisfaction in her clinical unit:

“When I came here, I thought I would be in a big city. But I came to this city. At first, I didn’t like the place. However, after joining the workplace, the cardiac surgery department, I liked it very much.”

(P1)

This shift illustrates how integration was continually reworked through accumulated workplace experiences. Viewed through Feldman’s model, these accounts suggest that movement toward change and acquisition was not uniform or complete; greater familiarity and confidence could develop alongside continuing relational, communicative, and organizational difficulties.

Across the four themes, the analysis therefore portrayed integration as a relational and experiential process that developed over time, while remaining uneven because access to organizational preparation, supportive relationships, and opportunities for workplace learning varied across participants and clinical contexts.

## 4. Discussion

This study explored how IRNs described workplace integration in Saudi healthcare settings through an organizational socialization lens. Their accounts show that integration did not depend on a single orientation event or on individual adaptation alone. It developed through the interaction of formal onboarding, everyday workplace relationships, repeated clinical exposure, and local unit practices. This interpretation is consistent with recent work describing workplace integration among migrant nurses and midwives as a multidimensional, time-dependent process involving socialization, communication, professional values, knowledge development, skill use, and participation [14]. In this discussion, workplace integration refers to effective participation and contribution within the clinical and organizational environment [14,15]. Professional belonging refers to feeling accepted and valued, whereas professional inclusion concerns equitable opportunities for recognition, voice, participation, and meaningful contribution [31]. These concepts overlap but are not interchangeable. An IRN may function effectively within a team while still feeling that her experience, judgment, or opportunities to contribute are insufficiently recognized.

### 4.1. Contextualizing Integration Within the Saudi Healthcare and Migration Environment

Participants’ experiences should be interpreted within the sociocultural and migration context of internationally recruited nursing in Saudi Arabia. All participants were women from India or the Philippines, two major nationality groups represented within the internationally recruited nursing workforce, which remains predominantly female [27,32]. Their workplace integration occurred in a healthcare system that continues to rely substantially on internationally recruited nurses while expanding national workforce participation [6,33]. It also took place in a multilingual clinical environment in which English commonly facilitates professional communication, while Arabic and culturally specific patterns of nurse–patient interaction remain important in everyday care [19,26]. Saudi research has also identified family separation, language and communication, remuneration, and professional expectations as salient aspects of expatriate nurses’ experiences [34].

Some of participants’ accounts were also situated within a period of substantial structural and workforce transformation in the Saudi health sector. Under Vision 2030 and the Health Sector Transformation Program, reforms have included changes in healthcare governance, expansion of health clusters, and greater attention to workforce capacity and sustainability [35]. National workforce planning likewise emphasizes workforce gaps, increasing Saudi participation in the health professions, and strengthening workforce capability [33]. Within nursing, national initiatives have sought to increase the number of qualified nurses and encourage Saudi participation in the profession [35]. For IRNs, integration therefore takes place within an evolving organizational and workforce environment. However, participants did not explicitly attribute perceived inequity, workplace support, or belonging to Vision 2030 or localization policies; these policies are therefore treated as contextual conditions rather than direct explanations of their experiences.

### 4.2. Fragmented Organizational Socialization Structures

From an OST perspective, the findings indicate an interdependent relationship between anticipatory socialization and the subsequent encounter stage. Limited workplace-specific preparation before entry left some participants with incomplete expectations regarding organizational roles, routines, and local workplace norms. This increased reliance on informal socialization mechanisms, particularly colleagues, supervisors, and experiential learning, to acquire workplace knowledge after entry. Where post-entry orientation was also brief, administratively focused and uneven across units, these earlier gaps were not fully addressed through formal organizational socialization.

This pattern suggests weakly structured anticipatory and encounter-stage socialization, with limited opportunities for role clarification, staged learning, and gradual transition into unit expectations [13,24]. Similar work has shown that the transition of IRNs is shaped by orientation quality, organizational and peer support, communication, job assignment, and workplace conditions [36], and that structured transition pathways can support entry into host healthcare systems [37]. In the present study, the practical consequence of fragmented formal support was that much of the work of learning shifted to IRNs and their immediate colleagues. Participants relied on personal initiative and whatever peer guidance was available to understand clinical practices, communication norms, and workplace expectations. Informal learning was often helpful, but it depended on the people and practices present in each unit. This helps explain why it supported day-to-day adjustment while also contributing to uneven integration experiences. The importance of peer and organizational support is consistent with international evidence on the social integration and acculturation of IRNs [4].

### 4.3. Relationally Mediated Integration

Workplace relationships were not simply sources of emotional support; they were also important channels through which participants learned how the organization worked. Colleagues, supervisors, and experienced nurses helped IRNs interpret clinical practices, language use, and cultural expectations that were not fully addressed through formal onboarding. Respectful guidance, patience, and approachability supported practical learning and a sense of belonging. This is consistent with qualitative evidence highlighting support, communication, valuing the individual, and inclusive organizational culture during the transition of IRNs [38]. In this study, everyday relationships therefore formed part of the integration process itself rather than merely compensating for gaps in onboarding.

These relationships were not always supportive. Some participants described blame, harsh responses to mistakes, and hierarchical pressure. These accounts can be interpreted in relation to the psychological safety literature [39,40], although psychological safety was not directly investigated or measured in this study. Rather, the findings indicate that the relational climate influenced how comfortable participants felt asking questions, expressing uncertainty, and seeking help. In less supportive environments, IRNs could remain functionally integrated within the team while becoming more cautious in their interactions and help-seeking behaviors.

Belonging adds another dimension to this relational picture. Participants who felt accepted, respected, and listened to described a stronger sense of membership, whereas inequitable or dismissive interactions could weaken that experience. Belonging, however, is not the same as inclusion. Inclusion also concerns whether an individual is valued for the knowledge and contribution she brings to the group [31]. An IRN may therefore feel socially welcomed but still perceive limited recognition, voice, or opportunity to contribute [40,41]. This distinction is useful when considering professional recognition without claiming that the present data demonstrate formal career exclusion. Access to supportive supervisors and experienced colleagues provided social resources through which participants learned workplace expectations, interpreted uncertainty, and developed a sense of organizational membership.

### 4.4. Professional Recognition and Skill Utilization as Broader Dimensions of Integration

This study did not directly explore professional recognition and specialty-skill utilization. However, wider literature suggests that limited recognition or use of prior expertise may constrain fuller workplace integration even when day-to-day adaptation occurs [41]. These issues are therefore considered broader dimensions of integration rather than findings of this study and warrant direct investigation in Saudi healthcare settings.

### 4.5. Communication Support and Workplace Learning

Communication challenges were woven through participants’ integration experiences. IRNs described learning Arabic words, adjusting interaction styles, and relying on colleagues to explain linguistic and cultural expectations. These difficulties reflected not simply individual language limitations, but the demands of working in multilingual and culturally specific clinical environments. Previous work suggests that structured transition education combining clinical placement, preceptor support, and targeted communication development can strengthen professional self-concept and transition [37,42]. In the present study, uncertainty about language and local interactional norms could also affect how confidently IRNs participated in clinical discussions and enacted their professional roles. Targeted support may therefore facilitate both day-to-day adjustment and fuller participation within clinical teams.

### 4.6. Experiential Internalization of Workplace Norms

Participants learned many professional and cultural norms through experience rather than through formal instruction. Observation, daily interaction, and repeated exposure helped them interpret communication practices, clinical routines, and expectations that were not always made explicit. This gradual learning became an important route to adaptation when formal preparation was limited. In this sense, experiential learning functioned partly as a compensatory socialization mechanism, addressing workplace knowledge and expectations that had not been fully established during pre-entry preparation or initial orientation.

Previous research on international nurses has described a similar pattern. Chun Tie et al. [43] portrayed integration as a progressive process of entering a new workplace, learning its cultural norms, becoming socialized through support, and adapting professional practice. The present findings add a caution to this experiential pathway: learning through experience can be effective, but it also places more responsibility on IRNs when structured guidance is weak. IRNs must then interpret unfamiliar expectations while simultaneously carrying out their clinical roles.

### 4.7. Divergent Integration Trajectories

Integration did not follow the same course for all participants. Some described growing confidence and satisfaction, whereas others continued to experience emotional strain, workplace pressure, or perceived inequity. These divergent trajectories can be interpreted as reflecting accumulated and interconnected socialization experiences rather than differences arising only during later adjustment. Variation in pre-entry preparation, orientation, relational support, and opportunities for workplace learning shaped the resources available to participants at successive stages of integration.

Prior research has similarly described integration as a phased process that requires support across different stages of adjustment [43]. Ramji and Etowa [15] emphasize that integration is a two-way process involving both organizational commitment and individual adaptation. In the present study, contrasting accounts indicate that respectful and equitable relational conditions may make adjustment easier, although these relationships were not tested. This interpretation is also consistent with research from Germany showing that perceived inequality was associated with lower workplace satisfaction among internationally recruited healthcare professionals [44].

### 4.8. Theoretical Contribution

The findings are consistent with several central propositions of organizational socialization theory while also illustrating how they may operate among IRNs. Feldman’s stages were useful for understanding the temporal pattern of participants’ experiences. Limited pre-arrival preparation reflected weaknesses in anticipatory socialization, while brief and inconsistent orientation characterized the encounter stage. Over time, participants described increasing familiarity with workplace expectations, communication practices, and organizational norms, consistent with processes of change and acquisition. However, this progression was neither uniform nor complete. Greater confidence sometimes coexisted with relational strain, communication challenges, and perceived inequities. For IRNs, socialization may therefore be recursive and uneven rather than strictly linear, with limitations in earlier stages increasing reliance on later relational and experiential learning. The findings therefore suggest that socialization stages are analytically distinguishable but empirically interdependent: limitations in anticipatory socialization altered the demands placed on encounter-stage support and increased the importance of later relational and experiential learning.

Van Maanen and Schein’s socialization tactics also help explain how formal and informal mechanisms interacted in practice. Formal orientation was frequently limited, while informal socialization through colleagues, supervisors, observation, and repeated workplace exposure became central to learning organizational expectations. Colleagues therefore functioned as socialization agents and informal role models, partially compensating for the absence of structured mentorship. In practice, formal organizational tactics and informal relational learning operated alongside one another but were not equivalent. Informal support can facilitate adaptation, yet its availability depends on local interpersonal relationships and may therefore produce uneven integration experiences.

These findings complicate the assumption that organizational socialization progresses through a consistently structured organizational pathway. Participants often developed functional competence despite fragmented formal socialization, suggesting that IRNs can acquire organizational knowledge through relational and experiential mechanisms outside formal onboarding. At the same time, reliance on these informal mechanisms transferred substantial responsibility for socialization from the organization to the IRNs and their immediate colleagues. The study adds contextual insight to OST by showing how organizational socialization among IRNs may be distributed across formal structures, workplace relationships, and experiential learning rather than being primarily organization-directed. Table 3 summarizes the relationships between the empirical findings and the relevant OST concepts.

### 4.9. Implications for Policy and Practice

The findings have practical implications for how hospitals organize the early employment of IRNs. A one-off orientation is unlikely to address the range of information, learning, and relational support described by participants. A staged pathway could begin before arrival and continue through early clinical practice, with accurate pre-arrival information, role-specific orientation, supervised transition, formal mentorship or preceptorship, communication support, and accessible supervisory feedback. In the present study, participants’ accounts suggest a need for more structured communication support within onboarding and mentorship. Accordingly, role-specific Arabic-English resources, designated preceptors, and scheduled supervisor check-ins are proposed as potential practice strategies. However, these strategies were not evaluated in this study, and their effectiveness requires further investigation. These approaches are consistent with evidence on structured transition education, communication development, supportive workplace relationships, and organizational environments that value IRNs [38,42]. Importantly, workplace integration should not depend primarily on the availability or goodwill of individual colleagues. Healthcare organizations should instead provide consistent structures for role clarification, mentorship, supervisory feedback, communication support, and equitable access to professional development. Such strategies should be evaluated empirically rather than assumed to work across settings.

### 4.10. Limitations and Future Research

This study provides a contextually rich account of workplace integration among IRNs in three public tertiary hospitals in southern Saudi Arabia, but several limitations should be considered. The all-female sample and concentration of Indian and Filipino participants limit the diversity of perspectives represented and may reduce transferability to male IRNs, nurses from other national backgrounds, other regions, and different healthcare sectors. The study was also limited to public tertiary hospitals within one region, and experiences may differ in private-sector, primary-care, or other organizational settings.

Data were collected at a single timepoint, while participants had worked in Saudi Arabia for at least two years and most had considerably longer experience. Participants entered the Saudi healthcare system at different points in time, spanning a period of substantial health-system transformation, workforce localization, and organizational change. Onboarding and integration practices may therefore have evolved during this period, meaning that accounts from participants with longer experience may reflect earlier organizational practices rather than those currently in place. Accounts of pre-arrival preparation and early organizational entry were also retrospective and may have been influenced by recall, subsequent professional experiences, and participants’ current perceptions of their workplaces. Although participants were encouraged to provide specific examples and clarify the context and sequence of events, recall bias cannot be excluded. A longitudinal design following IRNs from pre-arrival through later employment would provide a stronger account of how integration and organizational socialization develop over time.

The findings are based on participants’ subjective accounts and were not independently verified against organizational records, orientation materials, or the perspectives of managers, preceptors, or colleagues. Accordingly, descriptions of organizational support, perceived workplace inequity, and socialization practices should be understood as participants’ perceptions rather than objective assessments of organizational conditions. Future studies could incorporate multiple organizational perspectives and documentary sources to examine how integration processes are understood across stakeholder groups.

In addition, coding, theme development, and interpretation were undertaken by the author. Consistent with reflexive thematic analysis, no independent coding or inter-coder reliability procedure was used; however, the findings necessarily reflect the author’s interpretive positioning. Reflexive memoing, an audit trail, repeated engagement with the dataset, and attention to divergent accounts were used to enhance transparency in the interpretive process rather than to suggest that researcher influence could be eliminated. Participant validation, peer debriefing, and data triangulation were not used. Although these procedures are not methodological requirements of reflexive thematic analysis, their inclusion could have provided additional opportunities to consider the findings from alternative participant, researcher, or data-source perspectives.

Future longitudinal and comparative studies across units, institutions, regions, and healthcare sectors could examine how integration trajectories vary over time and identify organizational practices associated with more consistent integration. Research incorporating the perspectives of IRNs, managers, preceptors, and local colleagues could also provide a more comprehensive understanding of how prior expertise, belonging, voice, and organizational support are negotiated in practice.

## 5. Conclusions

Workplace integration among IRNs was a gradual and uneven process shaped by the quality of organizational socialization, workplace relationships, and experiential learning. Limited pre-arrival preparation, brief and non-sequential orientation, and inconsistent mentorship shifted substantial responsibility for learning workplace expectations onto nurses and their immediate colleagues. Informal relational and experiential learning often supported day-to-day adjustment, but because access to guidance depended on local relationships and unit practices, it did not provide a consistently structured pathway to integration. The findings suggest that healthcare organizations may benefit from treating integration as a sustained organizational process that begins before entry and continues through staged onboarding, mentorship, communication support, constructive supervisory relationships, and ongoing professional development. Strengthening these processes may reduce variability in integration and support the more effective participation of IRNs in culturally diverse healthcare teams.

## Figures and Tables

**Table 1:** Participant Characteristics (N = 19).

Characteristic	n	%
Age		
25–34 years	16	84.2
35 years or older	3	15.8
Gender		
Female	19	100
Country of Origin		
India	15	78.9
Philippines	4	21.1
Years of Experience in Saudi Arabia		
2–4 years	3	15.8
5–9 years	13	68.4
10 years or more	3	15.8
Employment Status		
Full-time	19	100
Part-time	0	0
Clinical Practice Area		
Critical and cardiac care	7	36.8
Perioperative services	6	31.6
Medical-surgical and specialty care	6	31.6
English Language Proficiency		
Low	0	0
Intermediate	4	21.1
Advanced or fluent	15	78.9
Arabic Language Proficiency		
Beginner	4	21.1
Intermediate or advanced	15	78.9

Note. Percentages are based on the total sample (N = 19). Language proficiency was self-reported. Age, years of experience in Saudi Arabia, and language proficiency are presented using the categories applied in the study dataset.

**Table 2:** Themes, Subthemes, and Analytical Descriptions.

Theme	Subtheme	Analytical Description
1. Entering the Workplace with Limited Organizational Preparation [n = 17]	1.1. Arriving with Limited Workplace-Specific Preparation [n = 12]	Participants entered the workplace with limited employer-provided information about clinical expectations, organizational norms, and role requirements, constraining anticipatory socialization before entry.
	1.2. Orientation as a Brief Gateway Rather Than a Staged Transition [n = 13]	Orientation was commonly brief and administratively focused, providing limited support for gradual role clarification, clinical adjustment, and structured organizational entry.
	1.3. Colleagues Filling the Mentorship Gap [n = 16]	In the absence of structured mentorship, colleagues and senior nurses became important informal socialization agents, providing practical, cultural, and workplace guidance during early adjustment.
2. Becoming Integrated Through Workplace Relationships [n = 19]	—	Workplace relationships shaped the relational conditions of integration, including support, acceptance, trust, belonging, vulnerability, and perceived inequity. Supportive interactions facilitated integration, whereas difficult relational environments constrained it.
3. Learning the Unwritten Workplace Through Experience [n = 18]	—	Participants acquired tacit workplace norms, communication practices, and organizational expectations through observation, repeated exposure, interaction, and everyday clinical participation rather than primarily through formal instruction.
4. Integration as an Uneven Trajectory Over Time [n = 15]	—	Integration developed as a cumulative but uneven trajectory, with increasing familiarity and confidence shaped by earlier preparation, organizational support, workplace relationships, and opportunities for experiential learning.

Note: Counts in square brackets indicate the number of participants, out of 19, whose coded material contributed to the corresponding final theme or subtheme. Participants could contribute to more than one theme or subtheme; therefore, counts are not mutually exclusive and should not be interpreted as measures of prevalence, frequency, or importance.

**Table 3:** Relationship Between Empirical Themes and Organizational Socialization Theory.

Empirical Finding	Relevant OST Concept	Theoretical Interpretation
Limited pre-arrival preparation	Feldman: anticipatory socialization	Participants entered with limited organization-specific expectations and preparation.
Brief and inconsistent orientation	Feldman: encounter; formal/informal and sequential/random socialization	Organizational entry was often weakly structured and inconsistently sequenced.
Colleagues teaching workplace practices	Socialization agents; informal and serial socialization	Experienced colleagues became informal socialization agents and role models when structured mentorship was limited.
Learning through repeated exposure	Informal socialization and experiential workplace learning	Organizational norms and expectations were acquired through observation, interaction, and participation rather than primarily through formal instruction.
Uneven integration over time	Feldman: change and acquisition; variable socialization	Adjustment developed over time but did not follow a uniform or linear trajectory.

Note: OST concepts were applied after theme development as interpretive resources and were not used as predetermined coding categories.

## Data Availability

Interview transcripts are not publicly available because the qualitative data may contain potentially identifiable information and are subject to ethical and confidentiality restrictions. De-identified data may be available from the corresponding author upon reasonable request, subject to appropriate ethical and confidentiality safeguards.

## Supplementary Materials

The following supporting information can be downloaded at: https://www.mdpi.com/article/10.3390/healthcare14203471/s1, Table S1, Participant Contributions to the Final Themes and Illustrative Quotations.

## Abbreviations

The following abbreviations are used in this manuscript:

IRN/IRNs	Internationally recruited nurse(s)
OST	Organizational Socialization Theory
PDOS	Pre-Departure Orientation Seminars
POEA	Philippine Overseas Employment Administration
SRQR	Standards for Reporting Qualitative Research
