# Supplementary material for: “My Colleagues Are the Ones Who Taught Me Everything”: Navigating Workplace Integration Through Organizational Socialization Among Internationally Recruited Nurses: A Qualitative Descriptive Study
Source: Healthcare (Basel). 2026 Oct 16;14(20):3471. doi: 10.3390/healthcare14203471 (PMC13704463; doi:10.3390/healthcare14203471)
Supplement: Supplementary file 1 [file healthcare-14-03471-s001.zip › healthcare-4605477-supplementary.pdf]

Supplementary Materials:

Table S1. Participant Contributions to the Final Themes and Illustrative Quotations

Participant	Theme 1	Theme 2	Theme 3	Theme 4	Illustrative quotation in Results
P1	✓	✓	✓	✓	Themes 1, 2, and 4
P2	✓	✓	✓		Theme 1
P3	✓	✓	✓		Theme 1
P4	✓	✓	✓		—
P5	✓	✓			Theme 2
P6		✓	✓	✓	Theme 3
P7	✓	✓	✓	✓	Themes 3 and 4
P8		✓	✓	✓	Theme 3
P9	✓	✓	✓	✓	—
P10	✓	✓	✓	✓	Theme 2
P11	✓	✓	✓	✓	Themes 1 and 4
P12	✓	✓	✓	✓	—
P13	✓	✓	✓	✓	Themes 1 and 4
P14	✓	✓	✓	✓	Theme 1
P15	✓	✓	✓	✓	—
P16	✓	✓	✓	✓	—
P17	✓	✓	✓	✓	—
P18	✓	✓	✓	✓	Theme 1
P19	✓	✓	✓	✓	Theme 2

Note. A check mark indicates that coded material from the participant contributed to development of the corresponding final theme. The final column identifies participants represented by illustrative quotations in the Results.

Participant contributions are presented to enhance analytic transparency and contextual interpretation and should not be interpreted as measures of theme prevalence, frequency, or importance. Detailed combinations of participant-level demographic and professional characteristics are not reported because, in this relatively small sample, such combinations could increase the risk of deductive disclosure or participant re-identification. Aggregate participant characteristics are reported in Table 1 of the main manuscript.
